# Metabolomics Reveals Discrimination of Chinese Propolis from Different Climatic Regions

**DOI:** 10.3390/foods9040491

**Published:** 2020-04-14

**Authors:** Tongtong Wang, Quanhui Liu, Min Wang, Limin Zhang

**Affiliations:** 1 Institute of Quality Standard and Testing Technology for Agro-Products, Chinese Academy of Agricultural Sciences (CAAS), Beijing 100081, China; wangttong123@126.com (T.W.); liuquanhui951010@126.com (Q.L.); 2 Key Laboratory of Agro-food Safety and Quality, Ministry of Agriculture, Beijing 100081, China; 3 Chinese Academy of Sciences (CAS) Key Laboratory of Magnetic Resonance in Biological Systems, State Key Laboratory of Magnetic Resonance and Atomic and Molecular Physics, National Centre for Magnetic Resonance in Wuhan, Wuhan 430071, China; zhanglm@wipm.ac.cn; 4 Wuhan Institute of Physics and Mathematics, CAS, Wuhan National Research Center for Optoelectronics, Wuhan 430071, China

**Keywords:** Chinese propolis, discrimination, geographic indicators, metabolomics, ^1^H NMR

## Abstract

The chemical profiles of propolis vary greatly due to the botanic sources and geographic origins, which limit its standardization for modern usages. Here, we proposed a reliable ^1^H NMR-based metabolomic approach, to discriminate the function and quality of Chinese propolis. A total 63 Chinese propolis samples from different temperate regions were collected and extracted for NMR analysis. Twenty-one compositions in ethanol extracts were assigned based on characteristic chemical shifts and previous literature reports. Significant geographic indicators were identified after the PCA and orthogonal partial least squares discriminant analysis (OPLS-DA) analysis of the obtained ^1^H NMR data. It was found that the composition discriminations arose from long-term acclimation of the different climates of botanic origin and caused the differences in the biological activities. This study provides us a reasonable instruction for the quality control of Chinese propolis.

## 1. Introduction

Propolis collected from plant bud resins and plant exudates is used for patching hives by worker bees. Since ancient times, it has been extensively applied in folk medicines, natural food additives, functional food ingredients, and natural cosmetics [1,2]. The propolis gathered from different plant origins and geographic origins, or produced by different species of bees, shows significantly different therapeutic properties, such as antimicrobial [3], anti-tumor [4], antioxidant [5,6], anti-inflammatory [7,8,9], immunomodulatory [10], anti-diabetic [11], and anti-ulcer properties [12]. These distinct activities are mainly attributed to the complicated chemical compositions of propolis [13,14,15], 50% of the content of which originates from plant resins [16]. Therefore, the discriminations of chemical compositions reflect adaptive response to diverse surroundings and climatic factors. It is an accumulated process acting on plants around the apiary. The chemical compositions can be considered as “a chemical link” between environments and biological activities [17,18]. The study of the variations of chemical compositions is of great importance to evaluate the diversity of biological activities from different climatic regions.

With the rapid development of “omics” technologies, metabolomics is a powerful and useful tool for carrying out a global analysis of all the metabolites [19]. Due to the deep insight into the compositional information, the concepts and research methods of metabolomics have been introduced to food nutritional evaluation [20], geographic origin identification [21], and classification [22]. Metabolomic profile data have been utilized to distinguish diverse propolis from different geographic origins. Numerous metabolomic research works on the chemical compositions of propolis by various techniques, including thin layer chromatography [23], high-performance liquid chromatography (HPLC) [24], ultra-high-performance liquid chromatography coupled with mass spectrometry [25], and gas chromatography coupled with mass spectrometry [26], have been reported. The NMR technology as a significant spectroscopic method with fast, robust, reproducible, and non-destructive features can provide the unbiased structural information of organic samples. Although it is less sensitive than chromatographic and mass methods, it is still a reliable and ideal tool for the qualitative and quantitative analysis of the entire composition. In addition, simple sample pretreatment, good stability, and short analysis time make it more suitable for high-throughput analysis. Recently, NMR-based metabolomics analysis was applied to a variety of propolis to study the characteristics and classification of southern Brazilian propolis [18], Nigerian propolis [24], as well as the discrimination of five regions: Asia, Brazil, Europe, Africa, and the Solomon Islands [27].

In the propolis industry, the mixture of different types of propolis from different regions without prior evaluation goes against the product descriptions. Besides, the lack of understanding of differences of the biological activities from diverse geographic origins does not benefit quality grading evaluation. Thus, the ability to determine the geographic origin of propolis products is conducive to promoting market regulation and quality control.

In the present study, we provide deep insight into the compositional differences of Chinese warm temperate propolis (CWTP) and Chinese mid-temperate propolis (CMTP) using ^1^H NMR spectroscopy to investigate the geographic origin-associated difference of the chemical compositions, coupled with multivariate statistical analysis. In addition, we discuss the geographic and surrounding conditions that result in the variations of the chemical compositions in two types of propolis. Based on the discussion, we give suggestions for the market regulation and industry standard revision.

## 2. Materials and Methods

### 2.1. Materials

All chemical reagents were of analytical grade. Solvents ethanol, ethyl acetate, and deuterated chloroform (CDCl_3_) were purchased from Merck, & Co. (Shanghai, China). NMR tubes were purchased from Wilmad LabGlass (Vinland, NJ, USA). Purified water was obtained by using a Milli-Q system (Millipore, Bedford, MA, USA).

### 2.2. Sample Collection and Preparation

**Figure 1:**
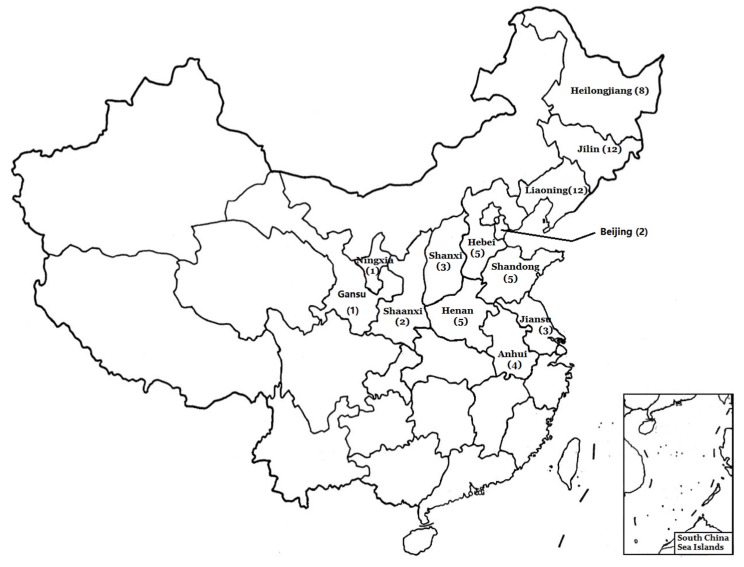
Distribution of sampling locations. The numbers in parentheses indicate the sample size of each province.

Sixty-three crude propolis samples were obtained from local beekeepers, collected from 13 different provinces belonging to two different temperate regions in China (Figure 1). All samples were produced by *Apis mellifera ligustica Spinola* and collected in July from 2017 to 2018. All the beehives were located in the plain. A total of 63 samples were collected, divided by the collection climatic regions as follows: the mid-temperate region, 32 samples; the warm-temperate region, 31 samples. Propolis was extracted from bee nests in a common woody hive box provided by Chinese beekeepers. The clamp was inserted under the hive’s cover, and the propolis were carefully scratched out, followed by the removal of wood debris and the dispersion of remaining bees. The qualities of samples were identified by professor Liming Wu. The voucher specimens were deposited in the laboratory of the Apicultural Research Institute. All the samples (~100 g/sample) were kept in plastic bags and stored at −20 °C until extracted.

Prior to extraction, the samples were crushed with mortars, homogenized, and then transferred into the plastic containers followed by flashing in nitrogen. Afterwards, each crude sample (100 mg) was dispersed into a 70% anhydrous ethanol solution (10.0 mL) and stirred vigorously at ambient temperature until the sticky solid was spread completely. The aqueous-EtOH emulsion was filtered to remove the flocculent deposit through the filter membrane (0.3 mm) after centrifugation. The filtrates were combined and concentrated by a rotary evaporator to afford the crude ethanol extracts. The extracts were dissolved with deionized water (10.0 mL) again and extracted three times with ethyl acetate (15.0 mL) by separating funnels to remove components with large molecular weights. These components would broaden the peaks for a lower T_2_ relaxation rate, and thus decrease the resolution.

**Figure 2:**
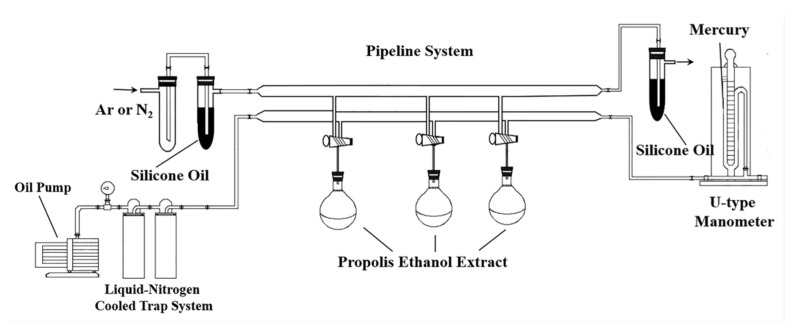
The Schlenk line used to remove water and organic solvent residuals.

Pulse sequences for solvent suppression such as noesypr-1d and zgpr are commonly applied to suppress the water peak. However, the blurring signals and the tilting baselines near the peaks of organic solvents and water cause errors in analyzing and integrating spectra. Therefore, the Schlenk line (Figure 2) was used to remove organic solvents and water residuals under reduced pressure using an oil pump at room temperature for 6.0 h. After distillation, argon was injected into round flasks to prevent sample oxidation. After dissolving in 0.70 mL CDCl_3_, each sample was transferred into 5 mm outer diameter NMR tubes for NMR analysis.

### 2.3. NMR Measurements

All samples were analyzed under identical instrument conditions and parameters. ^1^H NMR spectra were acquired on a 600.15 MHz Bruker Avance III spectrometer equipped with a BBI probe (Bruker Biospin GmbH, Rheinstetten, Germany) operating at ambient temperature. In the ^1^H NMR experiments, the pulse sequence zg was used with the acquisition parameters as follows: tuned, matched, and shimmed, 64 K data points each, 20.0 ppm spectral width, 2.5 s acquisition time, 3.0 s relaxation delay, and 64 transients. ^1^H NMR spectra were apodized by line broadening of 0.3 Hz before Fourier transformation. All spectra underwent baseline and phase calibrations by using Topspin 3.5 software. Tetramethyl silane (TMS) at δ 0.0 ppm was used for the chemical shift calibration. To facilitate NMR signal assignment, ^13^C NMR, ^1^H-^1^H COSY, TOCSY, ^1^H J-resolved, ^1^H-^13^C HSQC, and ^1^H-^13^C HMBC experiments were conducted for the selected sample to assign chemical compositions.

For the ^13^C NMR experiments, 10,240 transients were collected into 64 K data points. In both ^1^H-^1^H COSY and TOCSY spectra, 64 transients were acquired into 2 K data points for each of 128 increments. The MLEV-17 spin lock scheme was applied for TOCSY with a mixing time of 80 ms. The J-resolved experiments were employed to collect spin couplings with 64 transients into 8 K data points for each of 40 increments. For the HSQC experiments, the pulse sequence hsqcetgpsi was employed with composite pulse broadband ^13^C decoupling (globally alternating optimized rectangular pulses (GARP)) and 128 transients into 2 K data points for each of the 256 increments. For the HMBC experiments, the pulse sequence hmbcgpndqf was employed with 128 transients into 4 K data points for each of the 128 increments.

### 2.4. Quality Control

A reference sample of Chinese propolis (NIC-121324) with the known concentration of a component as quality control (QC) was designed to assess the recovery and the within-laboratory reproducibility of extraction (RSD_WLR_). Subsequently, aliquots of TMS were spiked to the extract the QC samples, which were used to measure the quantitative data by ^1^H NMR. Two aliquots of the QC sample were extracted each day during the trial. In total, 10 replicates of the QC sample were measured and quantified. All QCs underwent the same number of freeze/thaw treatments. The QC results showed that recovery and within-laboratory reproducibility were within the acceptability range (80–110% for recoveries, ≤15% for RSD_WLR_).

### 2.5. Data Preprocessing

In all samples, we chose the ^1^H NMR spectrum with the highest total spectral intensity for spectral alignment. Chemical shift misalignments in ^1^H NMR spectra of a complex biological matrix were common, probably arising from the instability of the magnet, the poor field homogeneity after shimming, the sample preparation process, and the sample itself. The adverse effect of migration led to errors and uncertainties in signal assignment and binning of ^1^H NMR data. An integrated software package named NMRSpec [28] was adopted to align all the spectra to adjust a tiny shift of the spectrum. Subsequently, the spectral region between 0.65 ppm and 8.50 ppm was binned/bucketed into 3690 integrated buckets with an equal width of 0.002 ppm. The signal region of the CDCl_3_ and highly overlapping resonance belonging to aromatic groups and solvent signals (7.20–7.50 ppm) were removed after the spectral alignment and baseline correction. To increase the data comparability of different samples’ concentrations, probabilistic quotient normalization (PQN) [29] was applied to reduce the interference of concentration variation by searching for the most probable dilution factor in Matlab software R2016b. PQN used the median of the quotients as the normalized standard to weaken the influence of outliers.

### 2.6. Statistical Analysis

Following data preprocessing, the normalized data were imported into SIMCA-P+ 14.1 (Umetrics, Umeå, Sweden) software for multivariate statistical analysis, including PCA and orthogonal partial least squares discriminant analysis (OPLS-DA). The Pareto scaling (Par) is considered as a useful compromise between the unit variance and the mean center scaling and can influence the results by valuable regions of low-medium amplitude. Therefore, the Par was applied to divide the normalized datasets by the square root of the standard deviation to decrease the differences among variables. PCA is an unsupervised pattern recognition analysis technique and used to reveal an overview of the clustering patterns of observations (samples). PCA transforms a set of possibly correlated variables into the less linearly uncorrelated variables to reduce the dimensionality and condense the dataset, simultaneously retaining the data integrity to the maximum extent. PCA can give any overall trend of the data independent of any prior knowledge, which may result in the expected discrimination. The interpretation of the PCA results is shown in the form of the score and loading plot.

OPLS-DA is used to further reveal the sample classification of chemical compositions and to explore the important variables responsible for discrimination. OPLS-DA applies the normalized NMR data as the X matrix and the class information as the Y variables. The validation of the model is conducted using a 7-fold cross-validation method. The quality of the model is defined by the parameters R2Y[cum] and Q2[cum], which are used to evaluate the cumulative amount of the explained variation for Y and the predictive ability. The OPLS-DA model was further assessed by CV-ANOVA tests for significant intergroup differentiations (at the level of *p* < 0.05).

To further illustrate the potential discriminating compositions, the correlation coefficient plot is drawn with the variable weights and color-coded with the absolute value of the correlation coefficient (abs (p(corr)). The variable weights were the back-scaled data from dividing loadings by the square root of the standard deviation. The cold color indicates the lesser significance of classification, while the hot color indicates the greater significance. The orientation of peaks implied the correlation between loadings and scores in OPLS-DA. The upward orientation in the correlation coefficient plot denotes a positive correlation to class discrimination and vice versa. The VIP values of discriminating compositions of interest showed the overall vital contributions of the variable (X) to all responses (Y) and were applied to further explain the differences among class. Moreover, the concentrations of discriminating compositions were determined separately by comparing the integral areas of characteristic peaks with those of the internal standard TMS in ^1^H NMR spectra. The significant intergroup discriminations between diverse compositions of two geographic propolis determined by quantitative ^1^H NMR spectra were examined by the Wilcoxon test with false discovery rate (FDR) correction in SAS (Version 9) and Matlab (Version R2016b) software. The statistical significance of the contents of the discriminating compositions was set at a *p*-value < 0.05 and <0.1, respectively.

## 3. Results

### 3.1. Assignment of ^1^H NMR Spectra

**Figure 3:**
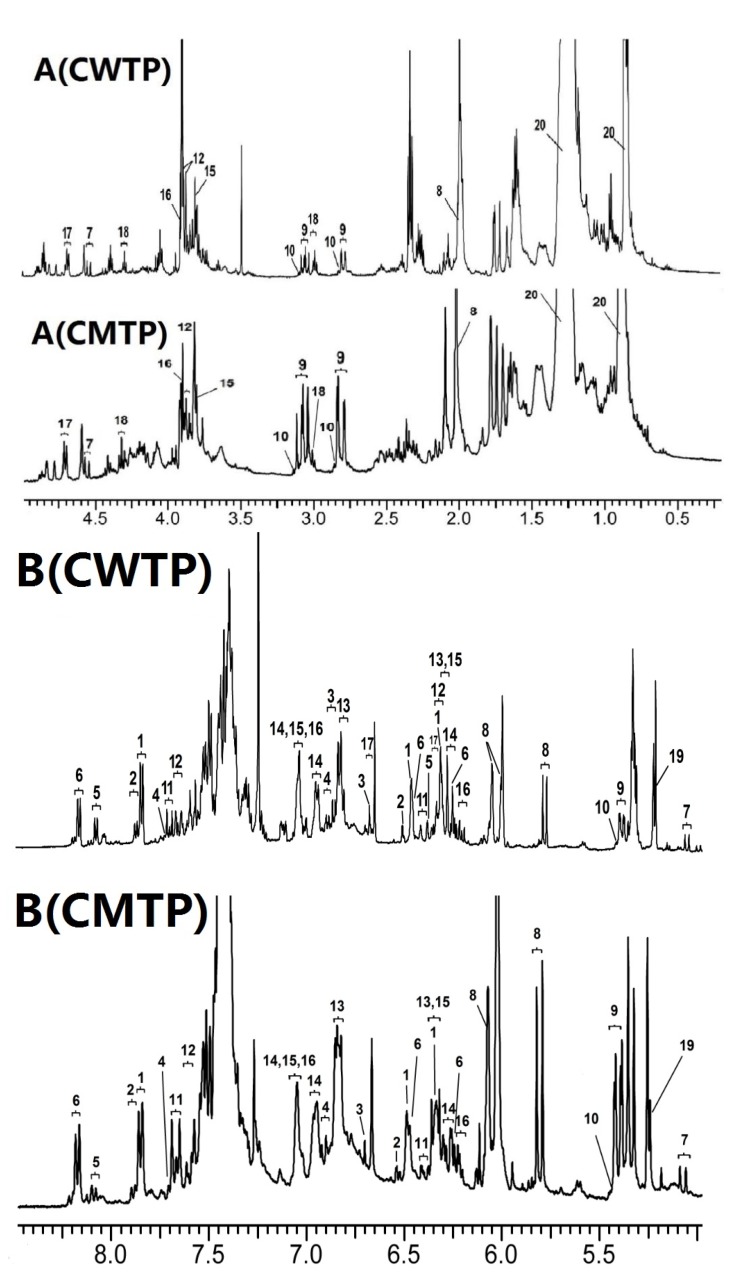
Typical 600 MHz ^1^H NMR spectra of ethanol extract of Chinese warm temperate propolis (CWTP) and Chinese mid-temperate propolis (CMTP). The chemical compositions are assigned and labeled with the numbers in Table S1. The spectrum is cut into two spectral regions: **A** (upfield, 0.20–5.00 ppm); **B** (downfield, 5.00–8.80 ppm).

As shown in Figure 3, the water single peak at 1.56 ppm and the ethanol quartet peak at 3.72 ppm in CDCl_3_ were effectively eliminated with the help of the Schlenk line. Meanwhile, the former research on HPLC profiles of Chinese propolis [13,14,15] was combined with NMR characteristic peaks to assign the main chemical compositions. The overall ^1^H NMR observation of 63 propolis samples was similar, indicating that all propolis samples originated from the same plant origin. Based on the previous research on the main origin of propolis in temperate regions [16], Chinese temperate propolis was termed as poplar-type propolis. The typical ^1^H NMR spectrum of samples is shown in Figure 3. The abundant chemical compositions in propolis have been well described in the literature of component analysis [16]. According to the elucidation of ^1^H NMR and TOCSY spectra, we identified 21 compounds from the ethanol extracts, including 10 flavonoids, nine phenolic acids and their esters, and other compounds (Table S1). Flavonoids primarily included 3 flavones, 3 flavonols, 2 flavanonols, and 2 flavanones. The main flavonoids were dominated by chrysin, pinocembrin, pinobanksin-3-O-acetate, and galangin, the relative content of which was consistent with HPLC profile analysis [13,14,15]. Flavonoids with a low level of concentration, such as tectochrysin, rutin, and myricetin, were not detected. The main phenolic acids and their esters contained cinnamic acid, caffeic acid, phenethyl caffeate (CAPE), and *iso*ferulic acid. The total content of phenolic acids and their esters in propolis samples was higher than that of flavonoids.

The obvious features of the compositions could still be noted in ^1^H NMR spectra. The strong signal upfield at around δ 1.25 ppm in ^1^H NMR spectra indicated still the existence of beeswax residues after careful pretreatments. It is noted that the signal in the range of δ 0.80–1.80 ppm of CMTP revealed much more aliphatic acid by comparison to the spectra of CWTP. Another closer inspection of the spectra showed that the spectral regions of CMTP and CWTP at δ 7.20–7.50 ppm were highly overlapped. The adverse effect of overlapped aromatic group signals in the downfield region could obscure the resonance of minor compounds, causing difficulty in identification. These phenyl signals were mainly contributed by common flavonoids and phenolic acids, and not regarded as characteristic signals. Therefore, this spectral region was removed from the variables after alignment.

Due to the similarity of flavonoid skeletons, errors in the assignment of components could be inevitable. However, compounds that possess unique signal peaks between δ 2.50 and 7.00 ppm could be used to make a distinction between the two types of propolis. The isolated CH2CH spin system in flavanone corresponded to the characteristic methylene signal peaks present as a doublet of doublets between δ 2.75 and 3.15 ppm. An enlarged view of the position at δ 5.08 and 4.56 ppm suggested the presence of two isolated methine protons in the flavanonol. The isolated 2, 3-trans double bonds in phenolic acids and their esters showed a characteristic doublet with 16.0 Hz *J*-coupling between δ 6.2 and 6.5 ppm. In addition, the two triplet signals at δ 3.00 and 4.31 ppm were related to the existence of CH2CH2 in CAPE, respectively.

### 3.2. PCA of ^1^H NMR Spectra

**Figure 4:**
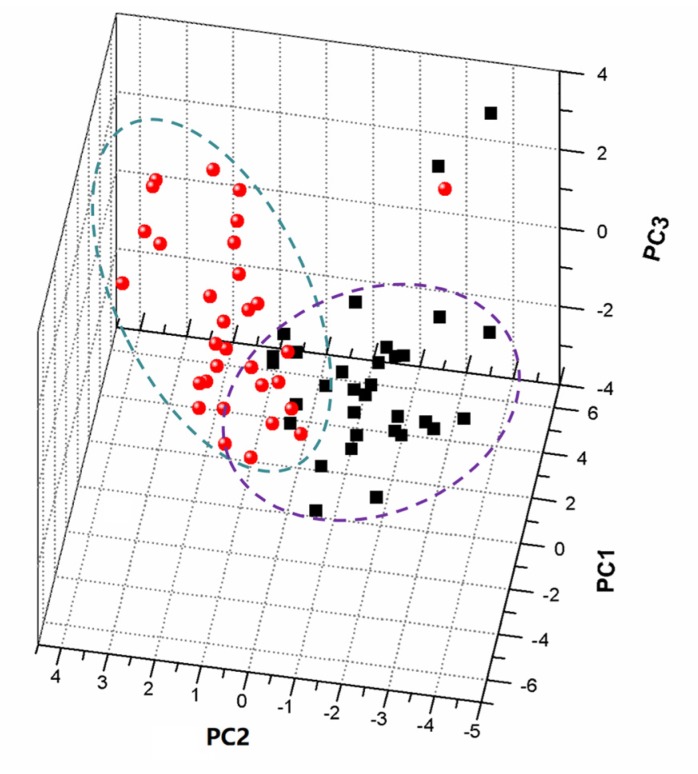
PCA score scatter plot of the ^1^H NMR data of CMTP and CWTP samples. The ellipse in scores represents the Hoteling T2 with 95% confidence.

Following the normalized dataset, PCA was applied to 3690 variables of 63 propolis samples to analyze the classification. Each observation (sample) could be represented by each point in the score plots, in which general trends and outliers could be explained visually. The loading scatter plot showed that the magnitude and manners of the variables were responsible for the behavior analysis of individuals. The overall trend of observations in a score plot was influenced by significant variables in the loading plot. The four principle components were extracted from the original data, accounting for over 76.8% of the data in the PCA model. In Figure 4, the PC1/PC2/PC3 score plot illustrates a partial separation between the CMTP and CWTP samples by PC1 of 32.8%, while a clear discrimination is observed in the direction of PC2 of 15.3% and PC3 of 10.2%. CMTP were placed at negative values between −4 and zero of PC2, while CWTP were found at positive values. Two CMTP outliers were observed outside the 95% confidence interval, and one CWTP sample deviated from the clustering of other CMTP samples. It was assumed that contamination of exogenous substances and debris led to the outlier result. In addition, the PCA model could retain the principle information, which was not necessarily important, due to there being no prior knowledge of the dataset. In the specific model, similar information of the chemical compositions could be extracted, while the potential difference was ignored.

### 3.3. OPLS-DA Models

**Figure 5:**
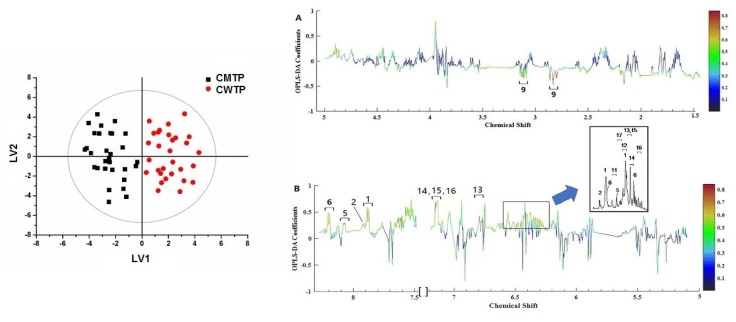
OPLS-DA scores (left) and coefficient-coded loadings plots (right **A** and **B**) for the models obtained from the ^1^H NMR data of CWTP and CMTP. The solid ellipse represents the Hotelling T_2_ with 95% confidence. The color bar corresponds to the correlation coefficient in the discrimination. The spectral region in the positive section (top half) is indicative of CWTP, while the negative half (bottom half) is indicative of CMTP. Cross-validated with CV-ANOVA, *p* = 5.57 × 10^−2^. Metabolite keys to the number are shown in Table S1.

OPLS-DA is a dimensionality reducing technique and a supervised model with discriminant analysis requiring knowledge of the dataset such as classification information. To achieve class discriminations, the spectral data and the distribution information were subjected to OPLS-DA. The variables of CMTP and CWTP are condensed into two principle components with R2Y[cum] 87.7% and Q2[cum] 48.7% in Figure 5 (left), indicating that these models were able to provide a good quality of separation. The results showed that the propolis in the warm temperate region collected in Hebei, Shanxi, Beijing, Henan, Shandong, Shaanxi, Gansu, Jiangsu, Ningxia, and Anhui provinces clustered together, and other propolis in the mid-temperate region gathered in Heilongjiang, Jilin, and Liaoning provinces clustered together. Meanwhile, variance testing of cross-validated predictive residuals (CV-ANOVA) further guaranteed the reliability of the OPLS-DA model at a level of *p* < 0.05.

**Figure 6:**
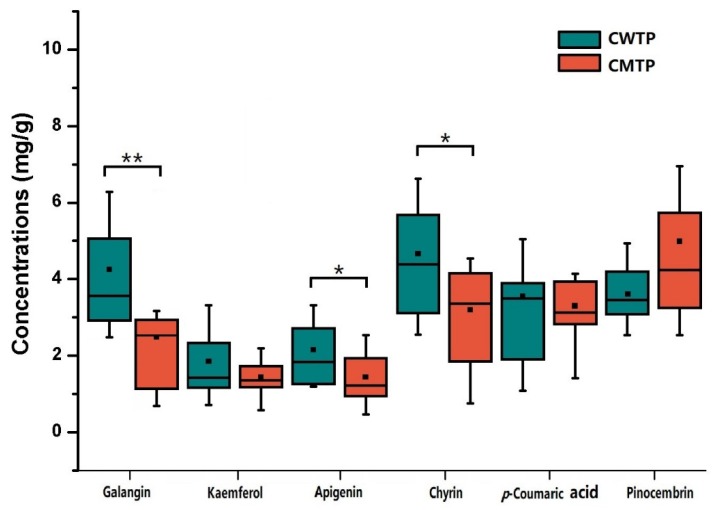
Box-plot graphic for the concentrations of the discriminating compositions in CWTP and CMTP for illustrative purposes. * *p* < 0.1, ** *p* < 0.05, Wilcoxon test with FDR correction.

To further reveal the discriminating compositions responsible for classification, the correlation coefficients and VIP values of important variables were evaluated (Table S2). The cut-off values were separately set based on the absolute value of the correlation coefficients (abs (p(corr)) [1]) >0.6 and VIP values >1.2, which are presented in Table S2. Combining with Figure 5, classification was positively correlated with the characteristic signals of CWTP arising from chrysin, apigenin, kaempferol, galangin, cinnamic acid, 3, 4-dimethoxycinnamic acid, *p*-coumaric acid, caffeic acid, ferulic acid, *iso*ferulic acid, and cinnamyl caffeate, while negatively correlated with the CMTP signals of pinocembrin. Because the characteristic peaks in the ^1^H NMR spectrum, such as cinnamic acid, 3, 4-dimethoxycinnamic acid, *p*-coumaric acid, caffeic acid, ferulic acid, *iso*ferulic acid, and cinnamyl caffeate, were susceptible to being overlapped with other compounds, affecting the accurate quantification, the concentrations of other discriminating peaks were calculated relative to the integral areas of TMS and further assessed by the Wilcoxon test with FDR correction (Figure 6). The results revealed that galangin was significantly higher (*p* < 0.05) in CWTP (4.11 ± 1.86 mg/g) compared to CMTP (2.54 ± 1.13 mg/g), while CWTP had higher chrysin (4.66 ± 2.00 mg/g) and apigenin (2.15 ± 0.78 mg/g) concentrations (*p* < 0.1) than those (2.95 ± 1.52 and 1.44 ± 0.75 mg/g) of CMTP. The Wilcoxon test with FDR correction showed that the concentration discrimination of kaempferol, pinocembrin, and *p*-coumaric acid (*p* > 0.1) between two the geographic propolis did not present obvious differences.

## 4. Discussion

In China, there are six different climatic regions, which are the tropical, the subtropical, the plateau, the warm temperate, the mid-temperate, and the cold-temperate regions in latitude-increasing order [30]. The *Populus* spp. as a common tree extensively grows in the mid-temperate, the warm temperate, and the subtropical regions [31]. Long-term acclimation to local climates and the surroundings causes variations of the levels of metabolites [32]. In general, prolonged spring drought, hot summer, and warm winter are the climatic characteristics of the northern China region, and the accumulated temperature per day is between 6.96 °C and 16.1 °C [33]. On the contrary, the northeast region is situated in the mid-temperate region with heavy spring floods, light spring droughts, cool summers, and frigid and short-solar radiating winters, and the accumulated temperature per day is between 4.73 °C and 6.96 °C [33]. In the coldest month, the mean temperature is below −10 °C with a long and frozen period for the rivers. The *Populus* spp. in the northern China region gains the strong ability of drought and high temperature resistance, while the species in the northeast China region has the obvious feature of cold resistance. The black soil in northeast China and the yellow soil in north China also differ in fertility [34]. There also exist obvious seasonal differences in the mean precipitation between north China and northeast China [35]. The changes in metabolic profiles reflect the interaction between the plants and the growing environments. The influence is an accumulative process building the genetic homogeneity.

Chinese propolis were reported mostly to have four biological activities, antioxidant [32], antitumor [14,36], anti-inflammatory [7] and neuroprotective activities [37], which were mainly attributed to the content and the ratio of polyphenols and phenols. As the changes in the local climate and surroundings cause variations of the chemical compositions, corresponding variations of the biological activities are also expected. The previous study on the biological activities of extracts showed obvious differences among propolis samples collected from different regions in China [38]. It showed that the strong antioxidant activity was contributed to the polyphenols and phenols by evaluating the DPPH radical scavenging, such as chrysin, 3,4-dimethylcaffeic acid, pinocembrin, galangin, pinobanksin-3-O-acetate, pinobanksin, *iso*ferulic acid, caffeic acid, CAPE, and kaempferol [38]. The contents of polyphenols and phenols in CWTP are greater than those in CMTP, implying that CWTP possesses more effective antioxidant activity than CMTP. In addition, it was found that water extracts of Chinese propolis contained more compositions, and thus displayed stronger antioxidant activity than ethanol extracts [13], indicating that the active compositions were easy to dissolve in water. Benzyl caffeate and CAPE were considered as significant compositions with strong cytotoxicity against colon 26-L5 carcinoma cell lines [14], which had similar contents in CMTP and CWTP, indicating that the climatic regions did not influence the cytotoxicity. Chrysin was known to have potential antitumor activity due to the enzyme inhibitory activity and the neuroprotective effect against neuronal cell death [37]. The level of chrysin was richer in CWTP than in CMTP, meaning that CWTP had greater antitumor activity than CMTP. The different biological activities of the poplar-type propolis were the result of the environment of the warm temperate region being more suitable for *Populus* spp. growth than that of the mid-temperate region.

According to the phytochemical research on propolis and plant bud resins [16], the bud exudate of *Populus* spp. was considered as a main source of Chinese propolis, except from Hainan province, Yunnan province, and Tibet. The phenolic profiles of ethanol extracts from Chinese propolis showed highly similar contents to that of resin from *Populus* canadensis *Moench*. Additionally, the study on the resin-collecting behavior of worker bees drew the same conclusion about plant origins [16]. The polyphenols and phenols with relatively high levels in CMTP and CWTP are chrysin, apigenin, pinobanksin-3-O-acetate, pinocembrin, galangin, and benzyl caffeate. These characteristic compositions are the almost the same as those of bud resin from *Populus canadensis Moench*, accounting for nearly 90% of total phenolic and polyphenolic types. Therefore, combining the biological activities, compositional differences, and relative contents, it was recommended that chrysin, galangin, and apigenin could be attributed to geographic indicators.

Currently, two industry standards are implemented to evaluate the propolis quality and authenticity in China. One industry standard (GB/T 24283-2009) suggests the total flavonoid content as a reference standard, which ignores the discrimination of the contents and biological activities from different geographic origins. Another industry standard (GB/T 19427-2003) makes a list of chemical compositions in Chinese propolis, including rutin, myricetin, quercetin, kaempferol, apigenin, pinocembrin, chrysin, and galangin. In the above lists, it was found that rutin, quercetin, and myricetin were not rich in Chinese propolis. Thus, we suggested that the evaluation standards could be adjusted to the chemical compositions with a high level of concentration, such as chrysin, apigenin, galangin, pinocembrin, kaempferol, 3, 4-dimethylcaffeic acid, caffeic acid, and *iso*ferulic acid. The results from this study could be used to improve the commercial production and consumption of Chinese propolis products.

Fundamentally, the climatic characteristics are vital factors that affect the biosynthesis of active compounds and, further, the biological activities of propolis samples [39]. The research on the discrimination of propolis is an indispensable part of the standardization of propolis product. The diverse climatic conditions of propolis were studied to establish the tight relationship between the biological activities of products and places of origin. A comparative study of botanical origins and chemical compositions of propolis from a wide range of countries revealed that the antibacterial activity was attributed to samples from locations featuring a wet-tropical rainforest-type climate [40]. The antioxidant and antibacterial activities of the Brazilian red propolis were influenced by climatic variations [41]. Due to the different climatic and vegetation characteristics, a distinct classification of Argentinian propolis between the coastal region and the top of the mountain region was shown by a digital image-based traceability tool [42]. The Brazilian south and southeast green propolis also revealed clear differences of phenolic compositions and bioactivities from the geographical and climatic conditions [43].

## 5. Conclusions

In summary, the present study demonstrated that multivariate statistical analysis on ^1^H NMR spectra is a useful and potential tool to evaluate compositional discrimination from two different geographic origins. Abundant information of ^1^H NMR has been used to achieve classification and rapid interpretation of compositional discriminations when combining with PCA and OPLS-DA analysis. The study highlighted that the correlation coefficients and VIP values could conveniently obtain the geographic indicators. The results of the study showed that the biological activities were closely related to the geographic locations and the climatic conditions. These findings could help the Chinese propolis industry re-evaluate the quality grading and authenticity of products.

## Supplementary Materials

The following are available online at https://www.mdpi.com/2304-8158/9/4/491/s1; Table S1: Assignment of chemical compositions in CWTP and CMTP. Table S2: OPLS-DA correlation coefficients p(corr) and VIP values of the discriminating compositions towards classification.
