# Supplementary material for: Metabolomics Reveals Discrimination of Chinese Propolis from Different Climatic Regions
Source: Foods. 2020 Apr 14;9(4):491. doi: 10.3390/foods9040491 (PMC7230208; doi:10.3390/foods9040491)
Supplement: Supplementary file 1 [file foods-09-00491-s001.pdf]

Metabolomics Reveals Discrimination of Chinese Propolis from Different Climatic Regions

Tongtong Wang [1,2], Quanhui Liu [1,2], Min Wang [1,2,*] and Limin Zhang [3]

[1] Institute of Quality Standard and Testing Technology for Agro-Products, Chinese Academy of Agricultural Sciences (CAAS), 100081 Beijing, P.R. China; wangttong123@126.com (T.W.); liuquanhui951010@126.com (Q.L.)

[2] Key Laboratory of Agro-food Safety and Quality, Ministry of Agriculture, 100081 Beijing, P.R. China;

[3] CAS Key Laboratory of Magnetic Resonance in Biological Systems, State Key Laboratory of Magnetic Resonance and Atomic and Molecular Physics, National Centre for Magnetic Resonance in Wuhan, Wuhan Institute of Physics and Mathematics, Chinese Academy of Sciences (CAS), Wuhan National Research Center for Optoelectronics, 430071 Wuhan, P.R. China; zhanglm@wipm.ac.cn

* Correspondence: wangmin@caas.cn; Tel: +86-010-82106545

Received: 12 February 2020; Accepted: 7 April 2020; Published: date

Table S1. Assignment of chemical compositions in CWTP and CMTP.

No.	Types	Identified compound	Structure of compound	Characteristic δ ^{1}H (ppm) and multiplicity	δ ^{13}C (ppm)
1		Chrysin	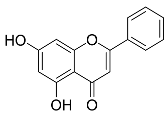	7.86 (d, J = 7.3 Hz,), 6.49 (d, J = 1.8 Hz, H-8), 6.34 (d, J = 1.8 Hz, H-6).	163.3 (C-2), 105.5 (C-3), 161.0 (C-5), 99.0 (C-6), 164.4 (C-7), 94.3 (C-8), 103.7 (C-10)，131.0 (C-1'), 126.4 (C-3' and 5'), 129.3 (C-2' and 6'), 132.0 (C-4').
2	Flavone	Apigenin	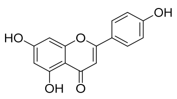	7.89 (d, J = 7.0, 1.2 Hz, H-2' and 6'), 6.53 (d, J = 2.0 Hz, H-8).	164.4 (C-2), 105.3 (C-3), 162.0 (C-5), 99.8 (C-6), 164.2 (C-7), 94.5 (C-8), 158.0 (C-9), 103.7 (C-10), 128.5 (C-2' and 6'), 116.0 (C-3' and 5'), 162.5 (C-4').
3		Luteolin	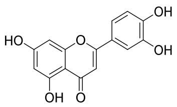	6.70 (s, H-3), 6.88 (d, J = 8.4 Hz, H-5').	164.2 (C-2), 105.5 (C-3), 162.0 (C-5), 99.0 (C-6), 164.5 (C-7), 94.3 (C-8), 158.0 (C-9), 103.7 (C-10), 113.1 (C-2'), 146.6 (C-3'), 150.1 (C-4'), 115.7 (C-5').
4		Quercetin	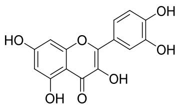	6.91 (d, J = 8.0 Hz, H-5'), 7.74 (dd, J = 8.0, 2.0 Hz, H-2').	146.6 (C-2)，135.1 (C-3), 161.5 (C-5)，98.4 (C-6), 163.7 (C-7), 94.5 (C-8), 156.0 (C-9), 102.9 (C-10), 122.4 (C-1'), 115.2 (C-2'), 145.1 (C-3'), 115.4 (C-5').
5	Flavonol	Kaempferol	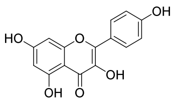	8.09 (d, J = 8.2 Hz, H-2' and 6'), 6.38 (d, J = 2.0 Hz, H-8).	146.7 (C-2), 136.1 (C-3), 161.5 (C-5), 98.4 (C-6), 163.1 (C-7), 93.5 (C-8), 103.1 (C-10), 122.4 (C-1'), 129.5 (C-2' and C-6'), 115.4 (C-3' and C-5'), 158.0 (C-4').
6		Galangin	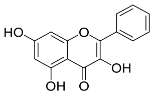	8.18 (d, J = 7.6 Hz, H-2' and 6'), 6.49 (d, J = 1.8 Hz, H-8), 6.28 (d, J = 1.8 Hz, H-6).	145.5 (C-2), 138.0 (C-3), 178.3 (C-4), 161.0 (C-5), 98.4 (C-6), 164.4 (C-7),

					93.6 (C-8), 157.0 (C-9), 102.4 (C-10), 122.5 (C-1'), 127.6 (C-2' and C-6'), 128.6 (C-3' and C-5'), 130.0 (C-4').
7	Flavanonol	Pinobanksin	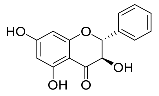	5.08 (d, *J* = 11.9 Hz, H-3), 4.56 (d, *J* = 11.9 Hz, H-2).	83.3 (C-2), 72.4 (C-3), 195.9 (C-4), 163.1 (C-5), 96.0 (C-6), 163.7 (C-7), 96.8 (C-8)，167.7 (C-9)，100.6 (C-10), 136.5 (C-1'), 127.6 (C-2' and 6'), 128.7 (C-3' and 5'), 129.1 (C-4').
8		Pinobanksin-3-O-acetate	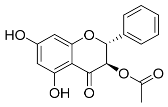	6.08 (d, *J* = 2.0 Hz, H-8), 6.03 (d, *J* = 2.0 Hz, H-6), 5.81 (d, *J* = 12.0 Hz, H-3), 2.03 (s, CH_3-3).	20.3 (CH_3), 168.1 (acetyl), 81.2 (C-2), 72.4 (C-3), 191.5 (C-4), 165.5 (C-5), 96.8 (C-6), 167.7 (C-7), 95.7 (C-8), 163.0 (C-9), 101.6 (C-10), 136.1 (C-1'), 128.0 (C-2' and 6'), 128.9 (C-3' and 5'), 129.5 (C-4').
9	Flavanone	Pinocembrin	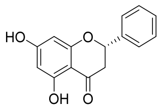	2.82 (dd, *J* = 17.2, 3.2 Hz, H-3), 3.09 (dd, *J* = 17.2, 13.1 Hz, H-3), 5.41 (dd, *J* = 13.1, 3.2 Hz, H-2).	79.2 (C-2), 43.3 (C-3), 195.8 (C-4), 165.5 (C-5), 96.8 (C-6), 167.7 (C-7), 95.2 (C-8), 164.2 (C-9), 102.9 (C-10), 138.3 (C-l'), 127.4 (C-2' and 6'), 129.1 (C-3' and 5'), 129.3 (C-4').
10		Pinostrobin	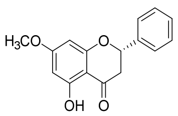	2,84 (dd, *J* = 17.2, 3.2 Hz, H-3), 3.11 (dd, *J* = 17.2, 13.1 Hz, H-3), 5.43 (dd, *J* = 13.1, 3.2 Hz, H-2).	55.7 (OCH_3), 79.2 (C-2), 43.2 (C-3), 195.6 (C-4), 164.2 (C-5), 95.2 (C-6), 168.1 (C-7), 94.0 (C-8), 162.8 (C-9), 103.1 (C-10), 138.3 (C-1'), 126.1 (C-2' and 6'), 128.9 (C-3' and 5'), 129.5 (C-4').
11	Phenolic acid	Cinnamic acid	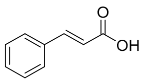	6.43 (d, *J* = 16.0 Hz, H-2), 7.73 (d, *J* = 16.0 Hz, H-1).	169.5 (C-1), 118.5 (C-2), 146.7 (C-3), 136.1 (C-1'), 129.5 (C-2' and 6'), 130.7 (C-3' and 5'), 131.0 (C-4').
12		3,4-Dimethox	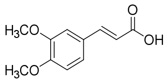	6.34 (d, *J* = 16.0 Hz, H-2), 7.68 (d, *J* = 16.0 Hz, H-1), 3.90 (s, OCH_3),	169.5 (C-1), 115.9 (C-2), 146.7 (C-3), 127.4 (C-1'),

		ycinnamic acid		3.92 (s, OCH_3).	110.5 (C-2'), 147.0 (C-3'), 156.7 (C-4'), 115.3 (C-5'), 124.1 (C-6'), 56.0 (OCH_3), 55.7 (OCH_3).
13		*p*-Coumaric acid	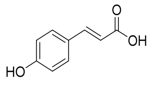	6.33 (d, J = 16.0 Hz, H-2), 6.85 (d, J = 8.0 Hz, H-3' and 5').	118.5 (C-2), 147.1 (C-3), 134.0 (C-1'), 128.4 (C-2' and 6'), 129.0 (C-3' and 5'), 130.8 (C-4').
14		Caffeic acid	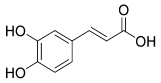	7.06 (d, J = 2.0 Hz, H-2'), 6.98 (dd, J = 8.0, 2.0 Hz, H-6'), 6.30 (d, J = 16.0 Hz, H-5').	115.4 (C-2), 147.0 (C-3), 127.7 (C-1'), 115.1 (C-2'), 149.4 (C-3'), 146.7 (C-4'), 116.5 (C-5'), 122.8 (C-6').
15		Ferulic acid	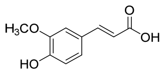	7.61 (d, J = 16.0 Hz, H-1), 6.33 (d, J = 16.0 Hz, H-2), 7.07 (dd, J = 8.0, 2.0 Hz, H-6'), 3.84 (s, OCH_3-3').	56.4 (OCH_3), 111.7 (C-2), 149.4 (C-3), 127.6 (C-1'), 116.6 (C-2'), 150.5 (C-3'), 147.1 (C-4')， 115.9 (C-5'), 124.1 (C-6').
16		*iso*Ferulic acid	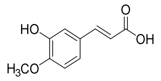	6.24 (d, J = 16.0 Hz, H-2), 7.07 (dd, J = 8.0, 2.0 Hz, H-6'), 3.93 (s, OCH_3-4').	55.7 (OCH_3), 167.7 (C-1), 122.4 (C-2), 144.1 (C-3), 127.3 (C-1'), 114.2 (C-2'), 146.6 (C-3'), 149.9 (C-4'), 113.0 (C-5'), 115.9 (C-6').
17	Phenolic acid ester	Cinnamyl caffeate	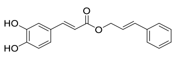	4.71 (t, J = 6.0 Hz, CH_2-1'), 6.71 (dt, J = 15.8, 2.1 Hz, H-3'), 6.40 (dt, J = 15.8, 6.0 Hz, H-2').	169.0 (C-1), 116.5 (C-2), 147.1 (C-3), 129.0 (C-1'), 114.9 (C-2'), 146.7 (C-3'), 149.6 (C-4'), 115.1 (C-5'), 123.0 (C-6' and 8''), 137.8 (C-1''), 127.7 (C-2'' and 6''), 129.6 (C-3'' and 5''), 124.6 (C-4''), 135.0 (C-7''), 66.0 (C-9'').
18		Phenethyl caffeate (CAPE)	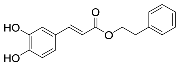	4.32 (t, J = 6.6 Hz, CH_2-1'), 3.02 (t, J = 7.0 Hz, CH_2-2').	167.7 (C-1), 114.2 (C-2), 145.2 (C-3), 127.6 (C-1'), 115.5 (C-2' and 5'), 146.5 (C-3'), 144.0 (C-4'), 126.6

					(C-6'), 138.3 (C-1''), 128.5 (C-2'' and 6''), 128.9 (C-3'' and 5''), 122.5 (C-4''), 36.3 (C-7''), 65.2 (C-8'').
19		Benzyl caffeate	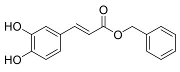	5.23 (s, CH_2-1').	167.7 (C-1), 122.4 (C-2), 144.1 (C-3), 126.4 (C-1'), 114.2 (C-2'), 145.4 (C-4'), 114.2 (C-5'), 115.3 (C-6'), 136.0 (C-1''), 128.3 (C-2'' and 6''), 127.7 (C-3'' and 5''), 121.6 (C-4''), 65.3 (C-7'').
20	Other compounds	Aliphatic acid	-	1.26 (brs, CH_2), 0.89 (t, $J = 7.2$ Hz, CH_3).	
21		Vanillin	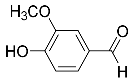	9.82 (s, CHO).	191.5 (CHO), 56.0 (CH_3O), 129.5 (C-1), 114.2 (C-2), 146.6 (C-3), 151.6 (C-4), 127.5 (C-6).

s, single; d, doublet; t, triplet; q, quartet; m, multiplet; dd, doublet of doublet; dt, doublet of triplet; brs, broad single.

Table S2. OPLS-DA correlation coefficients p(corr) and VIP values of the discriminating compositions towards classification.

No.	Component	Resonance (ppm)	p(corr)	VIP
1	Chrysin	7.86	+0.70	2.6
2	Apigenin	7.89	+0.75	1.3
5	Kaempferol	8.09	+0.81	1.2
6	Galangin	8.18	+0.63	2.2
9	Pinocembrin	2.82	-0.80	1.6
11	Cinnamic acid	6.43	+0.64	1.2
12	3,4-Dimethoxycinnamic acid	6.34	+0.72	2.6
13	*p*-Coumaric acid	6.85	+0.81	2.4
14	Caffeic acid	6.30	+0.70	1.2
15	Ferulic acid	6.33	+0.71	1.8
16	*iso*Ferulic acid	6.24	+0.65	1.5
17	Cinnamyl caffeate	6.71	+0.72	1.6
